# Supplementary material for: GM1 Oligosaccharide Crosses the Human Blood–Brain Barrier In Vitro by a Paracellular Route
Source: Int J Mol Sci. 2020 Apr 19;21(8):2858. doi: 10.3390/ijms21082858 (PMC7215935; doi:10.3390/ijms21082858)
Supplement: Supplementary file 1 [file ijms-21-02858-s001.pdf]

International Journal of *Molecular Sciences*

MDPI

Supplementary Information

GM1 oligosaccharide crosses the human blood-brain barrier in vitro by a paracellular route

Erika Di Biase[1◆], Giulia Lunghi[1◆], Margherita Maggioni[1*], Maria Fazzari[1], Diego Yuri Pomè[1], Nicoletta Loberto[1], Maria Grazia Ciampa[1], Pamela Fato[1], Laura Mauri[1], Emmanuel Sevin[2], Fabien Gosselet[2*], Sandro Sonnino[1] and Elena Chiricozzi[1*]

[1] Department of Medical Biotechnology and Translational Medicine, University of Milano, Milano, Italy; erika.dibiase@unimi.it (E.D.B.); giulia.lunghi@unimi.it (G.L.); maria.fazzari@unimi.it (M.F.); yuri.pome@gmail.com (D.Y.P.); nicoletta.loberto@unimi.it (N.L.); maria.ciampa@unimi.it (M.G.C.); pamela.fato@unimi.it (P.F.); laura.mauri@unimi.it (L.M.); sandro.sonnino@unimi.it (S.S.)

[2] Artois University, Blood-brain barrier laboratory, UR2465, Lens, France; emmanuel.sevin@univ-artois.fr (E.S.)

◆ These authors equally contribute to this work

* Correspondence: elena.chiricozzi@unimi.it (E.C.); marghemaggioni204@gmail.com (M.M.); Tel.: +39-02-503-30-380 (E.C.); fabien.gosselet@univ-artois.fr; Tel.: +33-321-791-733 (F.G.)

1. Methods

1.1. Cell authentication

1.1.1. N2a cells

N2a cells are not listed as a commonly misidentified cell line by the International Cell Line Authentication Committee. N2a cells were bought from Sigma-Aldrich to which they were supplied by European Collection of Authenticated Cell Cultures (ECACC) (Catalogue No. 89121404; Lot No. 13K010, passage +9). N2a were used from passage +10 to passage +15 to conduct experiments reported in the present manuscript.

To verify the authentication of employed N2a cells we performed the following tests at the beginning and end of single experimental work.

- Morphology check by microscope
  To identify the state of cells, we checked cellular morphology by phase contrast microscopy (Olympus BX50 microscope; Olympus, Tokyo, Japan). Morphological outcomes of N2a cells confirmed the expected neuronal/ameboid-like morphology (data not shown).
- Growth curve analysis
  Cell proliferation was evaluated according to MTT method [1, 2]. Briefly, 2.4 mM MTT (4 mg/ml in PBS) were added to each well and plates were re-incubated for 4 h at 37°C. Medium was carefully removed and replaced with 2-propanol: formic acid, 95:5 (v/v). Plates were gently agitated prior to read the absorbance at 570 nm with a microplate spectrophotometer (Wallac 1420 VICTOR2TM, Perkin Elmer). The growth profile showed a normal growth rate (data not shown).
- Mycoplasma detection
  Mycoplasma infection was evaluated by fluorescent Hoechst staining [3], a fluorescent dye that binds specifically to DNA and that reveals the presence of mycoplasma infections as intracellular particulate or filamentous fluorescence at 400X magnification using NikonEclipse Ni upright microscope. The mycoplasma test has always given negative results (data not shown).

1.1.2. Caco-2 cells

Caco-2 cells are not listed as commonly misidentified cell line by the International Cell Line Authentication Committee. However it is reported that culture condition and cell origin can deeply affect Caco-2 morphology and behavior [4-6]. Thus, we deeply verified and authenticated our Caco-2 cultures. We used Caco-2 cells during only ten passage (from passage +26 to +36) as previously reported [4].

- Morphology check by microscope
  We verified that our Caco-2 cells form compact and homogenous monolayer of mostly small diameter cells (data not shown).
- Mycoplasma detection
  Since mycoplasma could infect of Caco-2 cells, we validated the absence of any contamination by two methods (data not shown). As for N2a cells we directly staining nuclei of Caco-2 cells by fluorescent Hoechst staining. Moreover, we confirmed the absence of mycoplasma, by using the specific Lonza mycoplasma assay. Briefly culture supernatants were mixed with MycoAlert reagents according to manufacturer instruction and the luminescence was measured after 10 min using fluorimeter (BioTek, H1, Vermont, Winooski).

1.2. Morphological analysis for neurite outgrowth evaluation

N2a cells, treated or not with hBLEC-crossed OligoGM1 were observed by phase contrast microscopy (200X magnification, Olympus BX50 microscope; Olympus, Tokyo, Japan). At least 10 fields from each well were photographed for each experiment.

1.3. TrkA-ERK1/2 protein analysis

N2a cells grown in the presence or in the absence of hBLECs-crossed OligoGM1 were washed twice with 1 mM Na_3VO_4 and lysed with 1mM Na_3VO_4, 1 mM PMSF, 2% (v/v) aprotinin, and 1% (v/v) IP in cold PBS. Equal amounts of proteins derived from treated and untreated cells were denatured, separated on 4-20% precast polyacrylamide gels, and transferred to PVDF membranes using the Trans -Blot® Turbo™ Transfer System (Bio -Rad). The presence of TrkA, p-TrkA (Tyr490), ERK1/2 and p-ERK1/2 (Thr202/Tyr204) was determined by specific primary antibodies (diluted 1:1000 in 5% BSA in TBS-0.1% tween for TrkA, pTrkA and pERK1/2, while 1:2000 in 5% BSA in TBS-0.1% tween for ERK1/2), followed by reaction with secondary HPR-conjugated antibodies (diluted 1:2000 in 5% BSA in TBS-0.1% tween). α-tubulin was used as loading control. The data acquisition and analysis were performed using Alliance Uvitec (Eppendorf, Germany).

1.4. Evaluation of OligoGM1 toxicity on Caco-2 cells

Viability of Caco-2 cells after OligoGM1 treatments was determined by MTT assay (as previously reported [1,7]. Briefly, after 1 h from OligoGM1 incubation, cells plated in a 96-well were washed and incubated with 100 μL of 2.4 mM MTT (4 mg/mL in RH) for 1 h at 37°C in a humidify atmosphere of 95% air / 5% CO_2. Subsequently MTT was carefully removed and replaced with DMSO convert MTT into violet formazan. The quantity of formazan was measured by measuring the absorbance at 570 nm using a plate reading spectrophotometer (BioTek, H1, Vermont, Winooski) which was compared with a control condition (untreated Caco-2 cells) after cell solubilization with DMSO.

1.4. Establishment of permeability parameters

1.4.1. Apparent permeability (Papp)

Papp index is widely used to screen the absorption process of drugs [4, 8-10].
It is calculated according to the equation (1):

$$Papp = \frac{\left(\frac{\Delta Q}{\Delta t}\right)}{Co\ x\ S} \tag{1}$$

where (ΔQ/Δt) (μmol/sec) is the rate of permeation (quantity per second) of a molecule across the cell layer, C_0 (μmol/cm^3) is the donor compartment concentration at time zero and S (cm^2) is the area of the cell monolayer.

In the transwell insert system there are two compartments and, analyzing the absorption of a substance, the apparent permeability index can be obtained for the apical to basolateral transport, Papp A → B, and for the inverse, basolateral to apical transport Papp B → A.

To define Papp (1), the Fick's law could be used [11], which describes the transport of substances through a semipermeable membrane. Given that the initial concentration of the solute in the donor compartment is much higher than the one in the receiver compartment (equal to 0 at t_0), and assuming that the solute equally partitions in the two compartments, for small variation of C_0, it is possible to integrate and simplify the Fick's equation to obtain as follows:

$$\frac{\Delta Q}{\Delta t} = D\ x \frac{S}{h}\ x\ Co \tag{2}$$

where $\Delta Q/\Delta t$ corresponds to the amount of solute that crosses the membrane over time, D is the diffusion coefficient of the substance in water, S is the membrane surface, h its thickness and C_0 is the concentration of the solute which approximates the initial concentration C_0.

The equation (2) can be rewritten as:

$$\frac{D}{h} = \frac{\left(\frac{\Delta Q}{\Delta t}\right)}{S\ x\ Co} \tag{3}$$

equivalent to *Papp* (1), which can thus be defined as the ratio between D and the thickness h of the membrane. D is defined by the Stokes-Einstein [12] as follows:

$$D = \frac{k_B T}{6\pi\eta r} \tag{4}$$

where k_B is the Boltzmann constant, T is the absolute temperature and η the medium viscosity. This equation is particularly interesting because it relates the molecule mobility to its hydrodynamic *radius* (r).

Thus according to equations (1-4), the Papp of a solute directly depends on its hydrodynamic *radius* (r). Assuming that two different molecules at the same concentration move passively from one compartment to another, the relationship between their Papp at a given temperature defined in (4) will be equal to the inverse of the respective hydrodynamic *radius* values, as follows:

$$\frac{Papp\ A}{Papp\ B} = \frac{rB}{rA} \tag{5}$$

Using the equation above we can now directly compare the permeability of different molecules as long as their hydrodynamic *radius* are known.

1.4.2. Efflux ratio (E)

E value obtained applying the formula (2):

$$E = \frac{(Papp\ B > A)}{(Papp\ A > B)} \tag{6}$$

can be used to predict implication of active transport in the process of drugs absorption [13-19] Compounds with E > 2 are usually categorized as efflux transporter substrates, and on the contrary an E < 2 indicates a transport not mediated by transporters [20-22].

1.4.3. Endothelial permeability (Pe)

Pe is calculated to appreciate more accurately the transport of a drug across an endothelial barrier. In fact, this approach, based on the clearance principle, allows to obtain a result independent from the administered concentration [23, 24]. In order to obtain the increment in cleared volume between successive sampling events, the amount of solute transport during a time interval is divided by the donor chamber concentration. The total cleared volume is the sum of the incremental cleared volume up to the considered time points, according to the equation (6):

$$C = \frac{Q}{Cd} \tag{7}$$

where Q (pmol) is the amount of a drug in the receiver compartment and Cd is the donor chamber concentration at each time point (pmol / mm^3).

If during the experiment time, the clearance volume increases linearly, the average volume cleared can be plotted versus time. The slope, resulted by linear regression analysis of clearance, C (mm^3), and time, t (min), represents the permeability associated to a surface of transport:

$$Ps = slope\ (C\ x\ t) \tag{8}$$

The value can be obtained from endothelial cell-covered filters, PSt and from inserts covered only by Matrigel, PSf. Once elaborated this value, the endothelial permeability can be derived applying the equation (8):

$$\frac{1}{Pse} = \frac{1}{Pst} - \frac{1}{Psf} \tag{9}$$

and finally the following formula (9), that involves the measure of the transport surface, S (cm^2):

$$Pe = \frac{Pse}{S} \tag{10}$$

2. Supplementary Table S1

	LY Pe (10^{-3} cm/min)				
	0 μM	10 μM	50 μM	100 μM	300 μM
LY + GM1	0.65 ± 0.04	0.67 ± 0.017	0.56 ± 0.014	0.65 ± 0.012	0.63 ± 0.021
LY + OligoGM1	0.65 ± 0.04	0.61 ± 0.19	0.54 ± 0.021	0.69 ± 0.015	0.58 ± 0.017

Table S1. Direct transport related endothelial permeability (Pe A → B) (×10^{-3} cm/min) of LY (50 μM) alone or in combination with GM1 and OligoGM1 at different concentration (10, 50, 100 and 300 μM) after 60 min. Results are mean ± SEM from 4 independent experiments (*n* = 4) examining a minimum of 3 well for each condition.

3. Supplementary Figures

3.1. Supplementary Figure S1

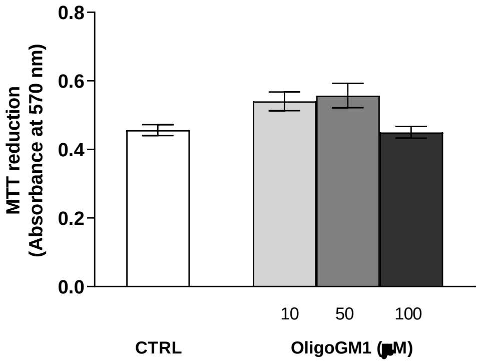

Figure S1. Effect of OligoGM1 on Caco-2 cell viability. Cell proliferation was evaluated by MTT reduction assay on Caco-2 cells in the absence or in the presence of different concentration of OligoGM1 (10, 50 and 100 μM) for 1 h. Results are expressed as mean of absorbance values at 570 nm ± SEM from 6 independent experiments (*n* = 6).

3.2. Supplementary Figure S2

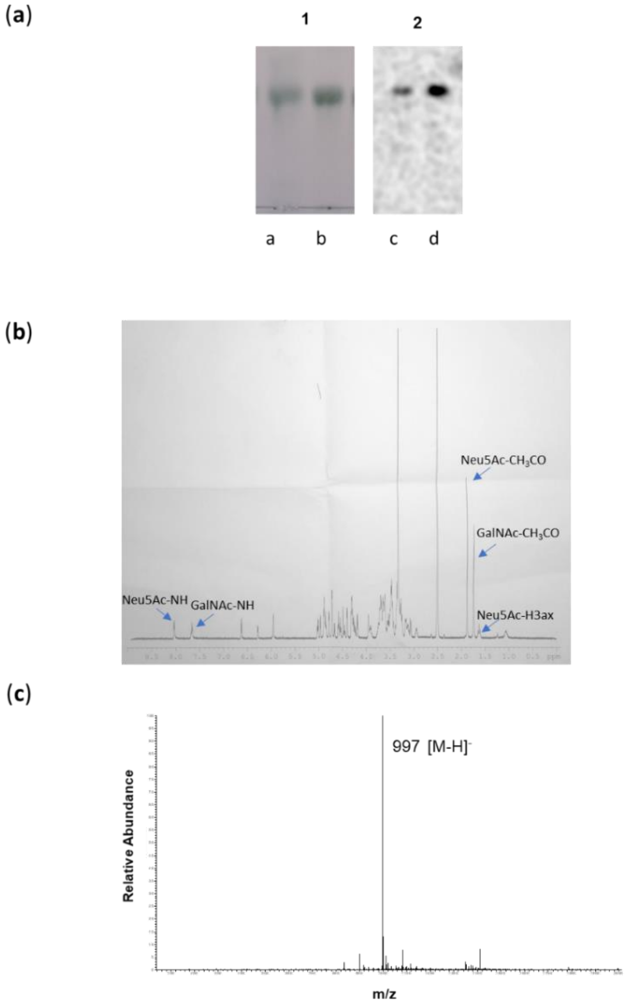

Figure S2. Analytical controls. (**a**) HPTLC. TLC were developed with the solvent system chloroform/methanol/KCl 50mM, 30:50:13 by vol. 1) Two amounts of OligoGM1, Erlich colorimetric revealing; 2) two amounts of [3H]OligoGM1, radioimaging TLC; (**b**) 500 MHz 1H-NMR spectrum in DMSO-d6 at 303K of OligoGM1; (**c**) ESI-MS (negative-ion mode): m/z= 997 [M - H]-.

3.3. Supplementary Figure S3

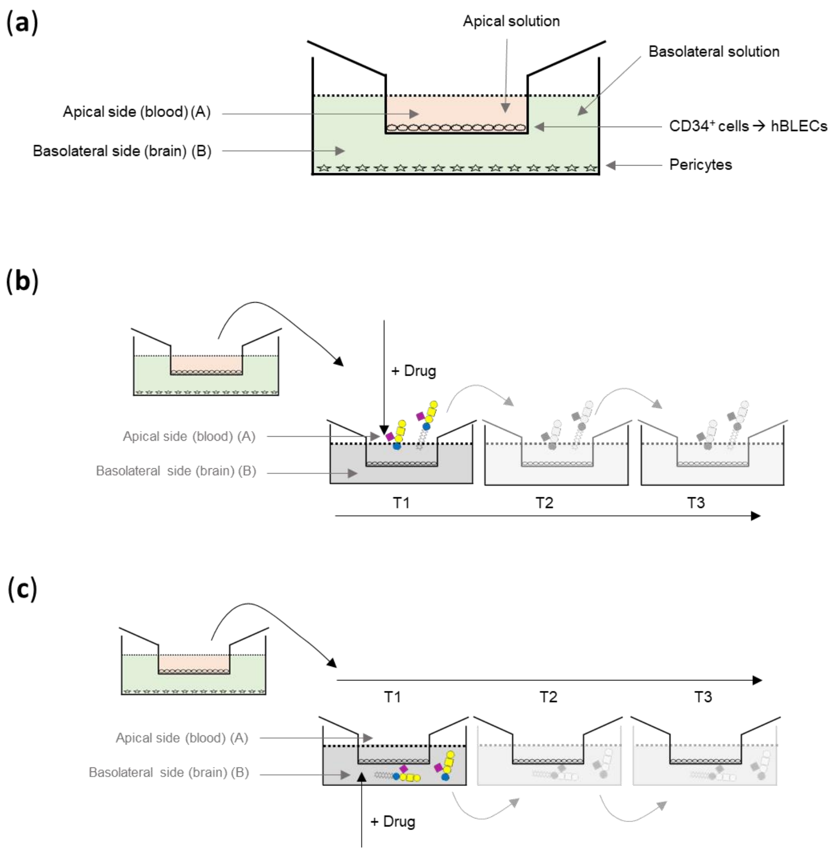

Figure S3. Schematic representation of the *in vitro* human BBB model. (**a**) General representation of the *in vitro* human BBB model. Human CD34⁺ cells are cultured on the apical side of a Matrigel-coated filter and brain pericytes are culture on the bottom of the well, in the basolateral side. After 6 days of co-culture CD34⁺ cells are fully differentiated into hBLECs, which can be used for the transport experiment between day 7 and day 20. The dotted line defines the level of the apical and basolateral solution volumes; (**b**) Apical to basolateral (A → B) transport experiment diagram. At time zero hBLECs-filter is transferred in a 12-well plate containing RH buffer and solution containing tested compound is added in the apical side, generally representing blood stream. At different time points, hBLECs-filter is moved into another well containing fresh RH buffer; (**c**) Basolateral to apical (B → A) transport experiment diagram. At time zero hBLECs-filter filled with RH buffer is transferred in a 12-well plate containing RH buffer plus tested compound. At different time points, a new hBLECs-filter is moved in the well containing the solution with tested compounds. GM1 sugar code is according to Varki *et al.* [25].

3.4. Supplementary Figure S4

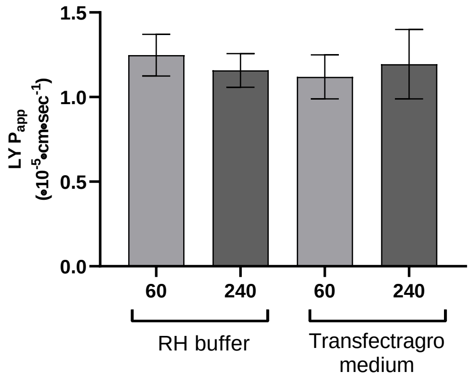

Figure S4. Direct transport related apparent permeability (Papp A → B) (×10^{-5} cm/sec) of LY (50 μM) after 60 and 240 min in RH buffer or Transfectragro medium. Results are mean ± SEM from 4 independent experiments (*n* = 4) examining a minimum of 3 wells for each condition.

References

1. Mosmann, T. Rapid colorimetric assay for cellular growth and survival: application to proliferation and cytotoxicity assays. *J Immunol Methods* **1983,** *65*, 55–63.
2. Chiaretti, S.; Astro, V.; Chiricozzi, E.; de Curtis, I. Effects of the scaffold proteins liprin-alpha1, beta1 and beta2 on invasion by breast cancer cells. *Biol Cell* **2016,** *108*, 65–75.
3. Aureli, M.; Bassi, R.; Prinetti, A.; Chiricozzi, E.; Pappalardi, B.; Chigorno, V.; Di Muzio, N.; Loberto, N.; Sonnino, S. Ionizing radiations increase the activity of the cell surface glycohydrolases and the plasma membrane ceramide content. *Glycoconj J* **2012,** *29*, 585–597.
4. Hubatsch, I.; Ragnarsson, E. G.; Artursson, P. Determination of drug permeability and prediction of drug absorption in Caco-2 monolayers. *Nat Protoc* **2007,** *2*, 2111–2119.
5. Anderle, P.; Niederer, E.; Rubas, W.; Hilgendorf, C.; Spahn-Langguth, H.; Wunderli-Allenspach, H.; Merkle, H. P.; Langguth, P. P-Glycoprotein (P-gp) mediated efflux in Caco-2 cell monolayers: the influence of culturing conditions and drug exposure on P-gp expression levels. *J Pharm Sci* **1998,** *87*, 757–762.
6. Hayeshi, R.; Hilgendorf, C.; Artursson, P.; Augustijns, P.; Brodin, B.; Dehertogh, P.; Fisher, K.; Fossati, L.; Hovenkamp, E.; Korjamo, T.; Masungi, C.; Maubon, N.; Mols, R.; Müllertz, A.; Mönkkönen, J.; O'Driscoll, C.; Oppers-Tiemissen, H. M.; Ragnarsson, E. G.; Rooseboom, M.; Ungell, A. L. Comparison of drug transporter gene expression and functionality in Caco-2 cells from 10 different laboratories. *Eur J Pharm Sci* **2008,** *35*, 383–396.
7. Sevin, E.; Dehouck, L.; Versele, R.; Culot, M.; Gosselet, F. A Miniaturized Pump Out Method for Characterizing Molecule Interaction with ABC Transporters. *Int J Mol Sci* **2019,** *20*.
8. Artursson, P. Epithelial transport of drugs in cell culture. I: A model for studying the passive diffusion of drugs over intestinal absorptive (Caco-2) cells. *J Pharm Sci* **1990,** *79*, 476–482.
9. Artursson, P.; Karlsson, J. Correlation between oral drug absorption in humans and apparent drug permeability coefficients in human intestinal epithelial (Caco-2) cells. *Biochem Biophys Res Commun* **1991,** *175*, 880–885.
10. Palumbo, P.; Picchini, U.; Beck, B.; van Gelder, J.; Delbar, N.; DeGaetano, A. A general approach to the apparent permeability index. *J Pharmacokinet Pharmacodyn* **2008,** *35*, 235–248.
11. Bokstein, B. S.; Mendelev, M. I.; Srolovitz, D. J. *Thermodynamics and Kinetics in Materials Science: A Short Course*. Oxford, **2005**; p 167–171.

12. Cruickhank Miller, C.; Walker, J. The Stokes-Einstein law for diffusion in solution. *Proc. R. Soc. Lond. A* **1924**.
13. Sugano, K.; Kansy, M.; Artursson, P.; Avdeef, A.; Bendels, S.; Di, L.; Ecker, G. F.; Faller, B.; Fischer, H.; Gerebtzoff, G.; Lennernaes, H.; Senner, F. Coexistence of passive and carrier-mediated processes in drug transport. *Nat Rev Drug Discov* **2010,** *9*, 597–614.
14. Molino, Y.; Jabès, F.; Lacassagne, E.; Gaudin, N.; Khrestchatisky, M. Setting-up an in vitro model of rat blood-brain barrier (BBB): a focus on BBB impermeability and receptor-mediated transport. *J Vis Exp* **2014,** *88*, e51278.
15. Uehara, I.; Kimura, T.; Tanigaki, S.; Fukutomi, T.; Sakai, K.; Shinohara, Y.; Ichida, K.; Iwashita, M.; Sakurai, H. Paracellular route is the major urate transport pathway across the blood-placental barrier. *Physiol Rep* **2014,** *2* (5).
16. Brasnjevic, I.; Steinbusch, H. W.; Schmitz, C.; Martinez-Martinez, P.; Initiative, E. N. R. Delivery of peptide and protein drugs over the blood-brain barrier. *Prog Neurobiol* **2009,** *87*, 212–251.
17. Lundquist, S.; Renftel, M.; Brillault, J.; Fenart, L.; Cecchelli, R.; Dehouck, M. P. Prediction of drug transport through the blood-brain barrier in vivo: a comparison between two in vitro cell models. *Pharm Res* **2002,** *19*, 976–981.
18. Kikuchi, R.; de Morais, S. M.; Kalvass, J. C. In vitro P-glycoprotein efflux ratio can predict the in vivo brain penetration regardless of biopharmaceutics drug disposition classification system class. *Drug Metab Dispos* **2013,** *41*, 2012–2017.
19. Stott, L. C.; Schnell, S.; Hogstrand, C.; Owen, S. F.; Bury, N. R. A primary fish gill cell culture model to assess pharmaceutical uptake and efflux: evidence for passive and facilitated transport. *Aquat Toxicol* **2015,** *159*, 127–137.
20. Summerfield, S. G.; Read, K.; Begley, D. J.; Obradovic, T.; Hidalgo, I. J.; Coggon, S.; Lewis, A. V.; Porter, R. A.; Jeffrey, P. Central nervous system drug disposition: the relationship between in situ brain permeability and brain free fraction. *J Pharmacol Exp Ther* **2007,** *322*, 205–213.
21. Polli, J. W.; Wring, S. A.; Humphreys, J. E.; Huang, L.; Morgan, J. B.; Webster, L. O.; Serabjit-Singh, C. S. Rational use of in vitro P-glycoprotein assays in drug discovery. *J Pharmacol Exp Ther* **2001,** *299*, 620–628.
22. Giacomini, K. M.; Huang, S. M.; Tweedie, D. J.; Benet, L. Z.; Brouwer, K. L.; Chu, X.; Dahlin, A.; Evers, R.; Fischer, V.; Hillgren, K. M.; Hoffmaster, K. A.; Ishikawa, T.; Keppler, D.; Kim, R. B.; Lee, C. A.; Niemi, M.; Polli, J. W.; Sugiyama, Y.; Swaan, P. W.; Ware, J. A.; Wright, S. H.; Yee, S. W.; Zamek-Gliszczynski, M. J.; Zhang, L.; Consortium, I. T. Membrane transporters in drug development. *Nat Rev Drug Discov* **2010,** *9*, 215–236.
23. Cecchelli, R.; Dehouck, B.; Descamps, L.; Fenart, L.; Buée-Scherrer, V.; Duhem, C.; Lundquist, S.; Rentfel, M.; Torpier, G.; Dehouck, M. P. In vitro model for evaluating drug transport across the blood-brain barrier. *Adv Drug Deliv Rev* **1999,** *36*, 165–178.
24. Siflinger-Birnboim, A.; Del Vecchio, P. J.; Cooper, J. A.; Blumenstock, F. A.; Shepard, J. M.; Malik, A. B. Molecular sieving characteristics of the cultured endothelial monolayer. *J Cell Physiol* **1987,** *132*, 111–117.
25. Varki, A.; Cummings, R. D.; Aebi, M.; Packer, N. H.; Seeberger, P. H.; Esko, J. D.; Stanley, P.; Hart, G.; Darvill, A.; Kinoshita, T.; Prestegard, J. J.; Schnaar, R. L.; Freeze, H. H.; Marth, J. D.; Bertozzi, C. R.; Etzler, M. E.; Frank, M.; Vliegenthart, J. F.; Lutteke, T.; Perez, S.; Bolton, E.; Rudd, P.; Paulson, J.; Kanehisa, M.; Toukach, P.; Aoki-Kinoshita, K. F.; Dell, A.; Narimatsu, H.; York, W.; Taniguchi, N.; Kornfeld, S. Symbol Nomenclature for Graphical Representations of Glycans. *Glycobiology* **2015,** *25*, 1323-1324.
